# Hypercaloric Diet Induces Cardiovascular Dysfunction Through Altered Purinergic Signaling on Atrial and Vascular Contractility

**DOI:** 10.3390/biology15141166

**Published:** 2026-07-16

**Authors:** Diego Castro Musial, Aron Jurkiewicz, Neide Hyppolito Jurkiewicz, Guilherme H. Souza Bomfim

**Affiliations:** 1 Department of Pharmacology, Paulista School of Medicine, Federal University of São Paulo (EPM/UNIFESP), São Paulo 04044-020, SP, Brazil; diego4630@gmail.com (D.C.M.);; 2 Department of Medicine, Federal University of Sergipe (UFS), Aracaju 49060-108, SE, Brazil; 3 Department of Physiology and Biophysics, Institute of Biomedical Sciences, University of São Paulo (ICB/USP), São Paulo 05508-000, SP, Brazil; 4 Department of Molecular Pathobiology, New York University College of Dentistry, New York, NY 10010, USA

**Keywords:** obesity, hypercaloric diet, purinergic system, atrium, aorta, cardiovascular

## Abstract

Hypercaloric diet and obesity are strongly associated with cardiovascular dysfunction, diabetes and hypertension development mediated by sympathetic nervous system hyperactivity. In addition to the adrenergic system, purinergic signaling has emerged as a relevant modulator of cardiovascular physiopathology. However, its role in hypercaloric diet-induced cardiovascular dysfunction remains incompletely understood. In this study, we investigated the effects of a hypercaloric diet on metabolic parameters, atrial purinergic modulation and vascular reactivity. Rats fed the hypercaloric diet consumed a diet with ~31% greater caloric density than control animals that received a standard (3.5 kcal/g) pellet diet for 8 weeks. Metabolic and cardiovascular parameters were measured, and isolated right atria (RA), left atria (LA) and thoracic aortic vessels were used to evaluate function, contractility and vascular responsiveness. A hypercaloric diet significantly increased body weight and visceral adiposity, with elevated systolic blood pressure (SBP) and cardiac hypertrophy, associated with alterations in glucose levels and insulin sensitivity. In isolated atria, a hypercaloric diet increased basal contractile force in the LA. Also, ATP induced a biphasic response characterized by an initial negative inotropic effect (NIE) followed by a positive inotropic effect (PIE). A hypercaloric diet reduced ATP-mediated NIE and enhanced PIE in both atria, suggesting an altered balance between inhibitory-P1 and excitatory-P2 purinergic signaling. At the vascular level, a hypercaloric diet impaired adenosine-mediated relaxation and increased contraction, indicating vascular hyperreactivity. Collectively, these findings suggest that a hypercaloric diet induces cardiometabolic dysfunction associated with alterations in cardiovascular function linked to impaired P1/P2 purinergic regulation, contributing to obesity-associated cardiovascular remodeling and hypertension.

## Simple Summary

Obesity caused by long-term consumption of hypercaloric diets is a major risk factor for cardiovascular diseases, diabetes, and hypertension. While excessive activation of the sympathetic nervous system is recognized as an important contributor to these disorders, the role of purinergic signaling, a pathway regulated by the extracellular molecule adenosine triphosphate (ATP), is less well understood. In this study, we investigated how a hypercaloric diet affects metabolic health, heart function, and blood vessel responsiveness in rats. Animals fed a hypercaloric diet developed obesity-associated metabolic dysfunction characterized by increased body weight, visceral fat accumulation, hyperglycemia, insulin resistance, elevated blood pressure, and cardiac hypertrophy. Functional studies demonstrated that the hypercaloric diet altered purinergic regulation in both the heart and blood vessels. In isolated atria, the balance between inhibitory and excitatory ATP-mediated responses shifted toward enhanced excitatory signaling, whereas isolated aortic rings exhibited impaired adenosine-mediated relaxation and increased vascular contractility. These findings indicate that hypercaloric diet-induced obesity disrupts cardiovascular purinergic signaling and contributes to cardiac and vascular dysfunction. Our study identifies purinergic pathways as potential therapeutic targets for preventing obesity-associated cardiovascular complications.

## 1. Introduction

The excessive consumption of hypercaloric and hyperlipidic diets promotes visceral fat accumulation, insulin resistance, glucose intolerance and metabolic and chronic inflammation, which contribute to cardiovascular impairment and a high risk of cardiometabolic diseases [1,2,3]. Modern dietary habits characterized by elevated intake of saturated fats, refined carbohydrates, ultra-processed foods and sugar-rich beverages have substantially altered metabolic homeostasis and increased the prevalence of obesity, insulin resistance, diabetes mellitus type 2 (DM2), hypertension and cardiovascular diseases (CVDs) [2,4,5,6]. Approximately ~50% of the world’s adult population (~2 billion people) are considered overweight or obese, with increases in obesity classes II and III [7,8,9]. Globally, it is estimated that eight countries represent ~54% of adults with overweight or obesity, including 172 million in the USA and Brazil with 88 million obese adults [8,9,10]. Overweight and obesity associated with hypercaloric diet habits increase the risk of developing CVDs by ~30–65% [11]. A high body mass index (BMI) accounts for ~10% of all cardiovascular-related deaths, with 2/3 of obesity-associated deaths being related to CVDs [12,13].

A hallmark of hypercaloric–hyperlipidic diets and obesity-related CVDs is an autonomic imbalance characterized by dysfunctional sympathetic nervous system (SNS) activity, which plays a central role in the development of obesity-associated heart and vascular alterations and hypertension [14,15,16]. Obesity is currently recognized as a chronic multifactorial disease associated with systemic metabolic dysfunction, chronic inflammation, endothelial dysfunction, autonomic imbalance and vascular remodeling [11,17]. Although the cardiovascular effects of sympathetic activation were primarily attributed to adrenergic neurotransmission mediated by α1-2-(α_1_-ARs) and β1-3-adrenergic receptors (β_1_-3-ARs), purinergic signaling is also an essential component of autonomic cardiovascular regulation [18,19]. Currently, it is well-established that purinergic signaling is a central regulator of cardiovascular homeostasis, autonomic neurotransmission, vascular tone and myocardial function mediated by adenosine triphosphate (ATP) coreleased simultaneously with norepinephrine from sympathetic nerve terminals [18,19,20,21].

Purinergic signaling is a key extracellular communication system mediated by nucleotides released from neurons, endothelial cells, cardiomyocytes and vascular smooth muscle cells (VSMCs) under physiopathological conditions [18,19,22]. ATP acts not only as an intracellular energy molecule but also as a neurotransmitter released simultaneously with norepinephrine from sympathetic nerve terminals, functioning as an important cotransmitter during autonomic activation [20,23]. Pharmacologically, purinergic receptors are divided into P1 and P2 families according to the extracellular ligand involved [24,25]. P1 receptors are activated primarily by adenosine, which is generated from ATP degradation through ectonucleotidases, composed of G-protein-coupled receptors (GPCRs) subtypes, A1, A2A, A2B, and A3 [24,25]. In the cardiovascular system, adenosine-mediated activation of A1 receptors exerts inhibitory and cardioprotective effects through G*i-o*-coupled signaling pathways, reducing intracellular cAMP levels, decreasing calcium (Ca^2+^) influx and attenuating sympathetic neurotransmission [24,25]. In atrial tissue, activation of A1 receptors promotes negative chronotropic and negative inotropic effects (NIE), reducing heart rate and contractile force [26,27]. Also, activation of A2A-B receptors stimulates vasodilatory pathways contributing to the maintenance of vascular compliance and blood flow regulation [26].

ATP/ADP, UTP and other nucleotides activate ionotropic P2X receptors and metabotropic P2Y receptors [19,25]. P2X (P2X1–P2X7) receptors are non-selective ligand-gated ion channels promoting membrane depolarization and intracellular Ca^2+^ influx after ATP binding, involved in VSMC contraction and sympathetic neurotransmission [28]. In contrast, P2Y receptors are coupled to G*q* and/or G*i* proteins, activating phospholipase C (PLC), inositol triphosphate (IP3), diacylglycerol (DAG), intracellular Ca^2+^ mobilization, and downstream kinase signaling pathways [29]. In cardiac atrial tissue, ATP produces a characteristic biphasic purinergic response, where the ATP degradation into adenosine promotes activation of inhibitory A1 receptors, resulting in a transient NIE [30]. Subsequently, direct ATP-mediated activation of excitatory P2 receptors induces a positive inotropic effect (PIE), associated with increased intracellular Ca^2+^ signaling and enhanced excitation-contraction coupling [30]. Thus, the balance between inhibitory P1-mediated and excitatory P2-mediated signaling is essential for physiological modulation of atrial contractility and autonomic cardiac regulation [19]. Similarly, in the aortic vessel, purinergic signaling plays a central role in the regulation of vascular tone and electromechanical coupling [18,30]. ATP released from sympathetic nerves and endothelial cells stimulates P2X and P2Y receptors in VSMCs, promoting vasoconstriction [19,29]. By contrast, adenosine-mediated P1 receptor activation induces vasorelaxation through NO-dependent endothelial pathways and reduction in intracellular Ca^2+^ signaling in VSMCs [19]. Therefore, alterations in purinergic signaling may contribute to vascular hyperreactivity, endothelial dysfunction, arterial stiffness and remodeling observed during hypercaloric diets, metabolic disorders and obesity-related CVDs [26,31].

Experimental and clinical reports suggest that metabolic stress alters extracellular ATP metabolism, purinergic receptor expression and adenosinergic signaling, contributing to vascular hyperreactivity, sympathetic overactivation and impaired cardiovascular homeostasis [31]. High-fat hypercaloric feeding in mice recapitulates key features of diabetic cardiomyopathy, while excessive dietary fat in rats promotes myocardial insulin resistance and cardiac functional impairment [32,33]. Metabolic stress and hypercaloric–hyperlipidic diets are associated with myocardial inflammation, fibrosis, apoptosis, insulin resistance, and contractile dysfunction [34]. Human studies indicate that purinergic receptor distribution or sensitivity may be altered in DM2, with impaired purinergic vasodilator responses contributing to endothelial dysfunction and reduced vascular perfusion [31]. P2X7 receptor signaling has been implicated in cardiac inflammatory and functional responses in high-fat diet models, whereas adenosine P1 receptor pathways are associated with cardioprotective, anti-adrenergic, and NIEs [29,35,36].

Although the functional impact of obesity-associated metabolic dysfunction on cardiovascular purinergic regulation remains incompletely understood, a shift from protective adenosinergic P1 signaling toward excitatory ATP-P2 purinergic signaling may contribute to these alterations by regulating sympathetic cotransmission. We hypothesized that chronic hypercaloric diet-induced metabolic dysfunction promotes functional remodeling of purinergic signaling, leading to altered atrial contractility and vascular reactivity. Therefore, the present study aimed to investigate the effects of a hypercaloric–hyperlipidic diet on metabolic parameters, atrial purinergic modulation, and vascular reactivity in rats, with particular emphasis on ATP-mediated inotropic responses and adenosine-dependent vascular relaxation. Our findings showed that a hypercaloric–hyperlipidic diet induced obesity-associated metabolic dysfunction by increasing body weight, visceral adiposity, hyperglycemia, insulin resistance, elevated systolic blood pressure (SBP) and cardiac hypertrophy. In isolated atria, a hypercaloric diet increased basal left atrial contractility and altered ATP-mediated purinergic responses by reducing NIEs and enhancing PIEs. Furthermore, a hypercaloric diet impaired adenosine-mediated aortic relaxation while increasing vascular responses, indicating vascular hyperreactivity and altered purinergic regulation associated with metabolic and cardiovascular dysfunction.

## 2. Materials and Methods

### 2.1. Animals and Hypercaloric Diet to Induce Obesity-Metabolic Alterations

All procedures employed in this study were conducted according to the guidelines of the Brazilian Association for Accreditation of Laboratory Animal Care (11.794/2008) and were approved by the Ethical Committee for the Care and Use of Animals. The experiments were approved by the Animal Research Ethics Committee from the Federal University of São Paulo (CEUA-EPM/UNIFESP), protocol number 66102102/14. Male Wistar rats aged 9.5–10 weeks (~230–250 g) obtained from INFAR (Instituto Nacional de Farmacologia e Biologia Molecular, EPM/UNIFESP), with a genetic background comparable to Charles River (Boston, MA, USA, strain code: #003), were used in this study. Animals were housed individually under controlled environmental conditions (23 ± 2 °C) relative humidity 55 ± 15% and a 12 h light/dark cycle, with free access to standard pellet-diet chow and water *ad libitum* throughout the experimental period. Rats were randomly assigned to two different experimental groups.

Hypercaloric Wistar rats received a hypercaloric diet with approximately ~31% greater caloric density through a palatable hypercaloric pellet diet compared with control animals that received a standard (3.5 kcal/g) pellet diet for 8 weeks. Briefly, rats from the hypercaloric group received a pelletized hypercaloric-hyperlipidemic diet designed to induce obesity and metabolic dysfunction through increased caloric density and high lipid content [37,38,39]. Hypercaloric pellet diets were industrially prepared by Nuvital^®^ (São Paulo, Brazil) with four alternating formulations (DH1–DH4), changed every 24 h to increase palatability and stimulate food intake. The hypercaloric pellet diets were supplemented with high-energy ingredients including roasted peanuts, corn oil, chocolate, wafer biscuits, potato chips, noodles, grated cheese, condensed milk and casein [39]. Compared with the standard chow rat diet, the hypercaloric diets exhibited increased lipid content and caloric density [39]. Standard rat chow contained approximately ~26% protein, ~54% carbohydrates, and ~3% lipids, providing 3.5 kcal/g. By contrast, the DH1–DH4 hypercaloric pellets were composed of ~26–28% protein, ~33–43% carbohydrates and ~20–24% lipids, providing ~4.6 kcal/g. This corresponds to a ~31% increase in caloric density compared with the control group diet. The elevated lipid content of the hypercaloric diets represented an approximately 7-fold increase relative to standard chow and was associated with the development of obesity-related metabolic alterations [39].

### 2.2. Body Weight, Visceral Adiposity and Cardiac Hypertrophy

Body weight was measured weekly from week 10 to week 20 in vivo throughout the experimental period using the same digital analytical balance. At the end of the protocol, animals were euthanized by decapitation, and visceral abdominal fat depots were carefully dissected and weighed to evaluate adiposity. The heart was rapidly excised, cleaned of connective tissue and blood residues, and weighed. Cardiac hypertrophy was evaluated by calculating the heart weight/body weight ratio (mg/g) [40].

### 2.3. Glucose Levels and Intravenous Insulin Tolerance Test (KITT)

Animals were fasted for 6 h before metabolic evaluation, and blood glucose levels were measured in vivo using blood samples collected from the tail vein with a glucometer system (Accu-Chek Active^®^, Roche Diagnostics, Basel, Switzerland). Insulin sensitivity was evaluated using the intravenous insulin tolerance test (ITT) [41,42]. Briefly, animals were anesthetized with sodium thiopental (50 mg/kg, i.p.) and received intravenous insulin injections (Novolin^®^, Novo Nordisk, Bagsvaerd, Denmark, 0.75 U/kg body weight). Blood glucose levels were measured at 0, 4, 8, 12, and 16 min after insulin administration. The rate constant for plasma glucose disappearance (KITT) was calculated, as we previously described [41,43].

### 2.4. Non-Invasive Blood Pressure Measurement

As previously described [40,44,45], SBP was measured weekly in conscious animals using in vivo non-invasive tail-cuff plethysmography (Mouse and Rat Tail Cuff Method Blood Pressure System; IITC Life Science, Woodland Hills, CA, USA). Prior to the experimental protocol, animals were acclimated to the procedure during a two-week adaptation period to minimize stress-associated artifacts. Measurements were performed under controlled environmental conditions and repeated until stable recordings were obtained.

### 2.5. Cardiovascular Functional Studies

The heart and thoracic aorta were rapidly removed and immersed in cold Krebs–Henseleit solution to perform ex vivo functional assays. Right atria (RA), left atria (LA), and thoracic aortic rings were carefully dissected and cleaned from connective and adipose tissues. Krebs–Henseleit solution contained (mM): NaCl 122.3, KCl 4.6, KH_2_PO_4_ 1.2, MgSO_4_·7H_2_O 1.2, NaHCO_3_ 17.4, CaCl_2_·H_2_O 1.5, and glucose 11.1. Solutions were continuously bubbled with a carbogen mixture (95%-O_2_ and 5%-CO_2_) at 37 °C and pH 7.4. Thoracic aortas were sectioned into 4 mm ring segments and mounted in isolated organ baths for isometric tension recordings. Aortic rings were maintained under a resting tension of 1.5 g and equilibrated for 60 min with solution replacement every 15 min before experimental protocols. Endothelial integrity was evaluated by acetylcholine-ACh-induced relaxation (1 μM) after pre-contraction with phenylephrine-Phe (1 μM). Only preparations exhibiting ≥80% relaxation were included in the study. Mechanical activity was recorded using force transducers (FT202, CB Sciences, San Diego, CA, USA) coupled to an amplifier system (ETH-400, CB Sciences, San Diego, CA, USA) connected to a PowerLab analog-to-digital acquisition system (AD Instruments, Colorado Springs, CO, USA). Data acquisition and analysis were performed using LabChart software version 5.1.2 (AD Instruments, Dunedin, New Zealand). The contractions were measured isometrically and recorded by force transducers, as we previously described [46].

### 2.6. Isolated Atrial Contractility

Isolated atria were immediately mounted in organ baths for ex vivo isometric contraction recordings. RA was allowed to beat spontaneously due to the presence of sinoatrial node automaticity [47]. By contrast, LA was electrically stimulated using platinum electrodes under field stimulation conditions (2 Hz frequency, 5 ms pulse duration). After mounting, atrial preparations were stabilized for 40 min before experimental protocols. Basal atrial contractility was evaluated in both RA and LA preparations. ATP-induced purinergic modulation was evaluated using ATP (100 μM). ATP induced a characteristic biphasic inotropic response consisting of an initial NIE, followed by a PIE. NIE was calculated as the percentage reduction relative to basal contraction force. PIE was calculated as the difference between the minimum contractile force observed during NIE and the maximal contractile response following ATP administration, normalized to basal contractility values. Results were expressed as delta (Δ) percentage (%) relative to basal contraction force.

### 2.7. Vascular Reactivity in Isolated Aortic Rings

After equilibration, aortic rings were pre-contracted with phenylephrine (1 μM). Endothelium-dependent relaxation was evaluated using acetylcholine-ACh (1 μM). Purinergic-mediated vascular relaxation was evaluated through cumulative concentration–response curves to evaluate P1-mediated vascular relaxation. Vascular relaxation responses were constructed from nano- (0.1 nM) to millimolar (1 mM) concentration–response plots for adenosine, expressed as a percentage reduction relative to phenylephrine-induced contraction. Electromechanical coupling and depolarization-induced contraction were evaluated using KCl (80 mM). Contractile responses were continuously recorded during KCl stimulation. Vasoconstrictor responsiveness was assessed using cumulative concentration–response curves to angiotensin II (0.01 nM to 10 μM). Contractile responses were normalized to maximal contraction values.

### 2.8. Data Analyses and Statistics

All data, mathematical analyses and graphs were analyzed and/or generated using the GraphPad Prism software version 10.5.0 (GraphPad Software, Boston, MA, USA), as we previously described [48,49,50,51]. Data are presented as mean ± SD of four to five (4–5) independent experiments; each individual dot in the boxplots and histograms represents a sample from an independent experiment. Concentration–response (0.1 nM to 1 mM) curve plots of adenosine were constructed using the sigmoidal log(inhibitor) vs. response-variable slope (four parameters) equation fitted by GraphPad Prism. The IC50 of adenosine was calculated, the concentration that provokes a response halfway between the basal (peak) response and the maximal inhibition (bottom) response. The parameter of the LogIC50 (apparent affinity) was obtained and fitted by the nonlinear regression curve. The sigmoidal log (inhibitor) vs. response-variable slope (four parameters) equation has the following function model: Y = Bottom + (Top-Bottom)/(1 + 10^((LogIC50-X)*HillSlope)). Statistical significance was determined using a two-tailed unpaired Student’s *t*-test, with significance indicated by *p* values. Significance was accepted as indicated by *p* values in boxplots and histograms. Symbols (*) and their respective *p* value were detailed in the caption of each figure.

## 3. Results

### 3.1. Hypercaloric Diet Induces Metabolic Dysfunction, High Blood Pressure and Hypertrophy

**Figure 1:**
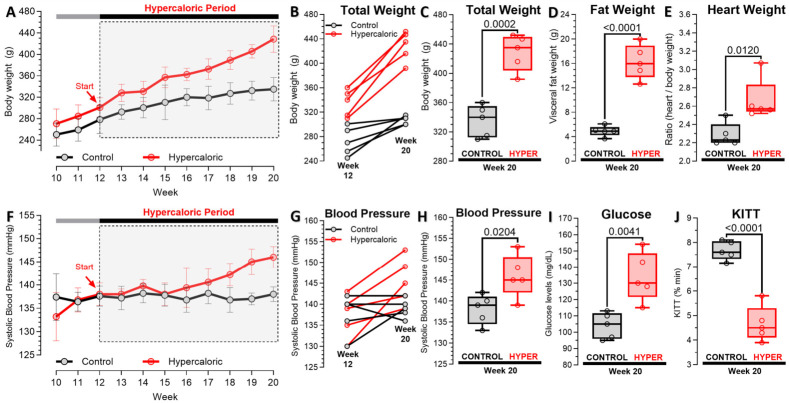
**Hypercaloric/hyperlipidic pellet diet induces obesity-associated metabolic dysfunction, hypertension and cardiac hypertrophy.** (**A**) Weekly body weight progression during the hypercaloric pellet-diet protocol, from week 10 to week 20 indicated by the red arrow, throughout the experimental period using a digital analytical scale. Individual paired analysis. (**B**) Total body weight (**C**) and visceral fat accumulation weight at week 20 (**D**)**,** comparing hypercaloric diet rats to control animals that received a standard pellet diet for 8 weeks. (**E**) Heart weight normalized by heart weight/body weight ratio (mg/g). (**F**) Systolic blood pressure (SBP) measurements throughout the hypercaloric pellet-diet protocol, with individual paired analysis (**G**) and SBP values at week 20. (**H**) Compared to the control standard pellet diet. Glucose metabolism was evaluated by fasting blood glucose levels (**I**) and intravenous insulin tolerance test (KITT) (**J**), demonstrating impaired insulin sensitivity in rats exposed to a hypercaloric pellet diet. Data are presented as mean ± SD from four and five independent experiments (*n* = 4 and *n* = 5), with data points shown for the control group (black) and hypercaloric group (red) as individual dots in the boxplots and histograms. Statistical significance was determined using a two-tailed unpaired Student’s *t*-test, with significance indicated by *p* values. Significance was accepted as indicated by *p* values.

Animals subjected to a hypercaloric diet progressively developed an obesity-associated metabolic and cardiovascular phenotype (Figure 1). Weekly body weight monitoring demonstrated a marked increase in body mass from the beginning (week 12), which remained elevated throughout the experimental period (week 20), compared with control animals (Figure 1A). Although body weight increased with age in both groups, paired analysis further confirmed a significant increase (~28%) in total body weight gain in hypercaloric animals compared with the control group at week 20 (Figure 1B,C). Visceral adiposity was markedly elevated in hypercaloric animals, with visceral fat accumulation increased by ~3.1-fold compared with control animals (Figure 1D), indicating successful induction of diet-induced obesity. Next, to address whether 8 weeks of a hypercaloric diet affect the metabolism and cardiovascular system, we measured in vivo and ex vivo cardiovascular and glucose metabolic parameters (Figure 1E–J). A hypercaloric diet promoted cardiac remodeling characterized by increased (~350 mg) heart weight, suggesting the development of cardiac hypertrophy (Figure 1E). In vivo cardiovascular blood pressure evaluation demonstrated that a hypercaloric diet also induced progressive increases in SBP. Weekly measurements showed that a hypercaloric diet elevated SBP in week 20 compared to control animals (Figure 1F). Individual paired analysis confirmed an increase in SBP between weeks 12 and 20 in hypercaloric rats (Figure 1G). Compared with the control group, in week 20, the hypercaloric diet increased the SBP by ~7–8 mmHg (Figure 1H). In addition, the metabolic evaluation demonstrated that a hypercaloric diet impaired glucose homeostasis and insulin sensitivity (Figure 1I,J). Rats exposed to the hypercaloric diet showed significantly increased (~25 mg/dL) fasting blood glucose levels compared with control animals (Figure 1I). Also, the insulin tolerance test analysis revealed a marked reduction (~3.0% min) in KITT values, indicating impaired insulin sensitivity and development of insulin resistance in hypercaloric animals (Figure 1J). Taken together, these findings demonstrate that an 8-week hypercaloric diet induces a cardiometabolic dysfunction, obesity phenotype, insulin resistance, hyperglycemia, increased blood pressure and cardiac hypertrophy that can affect the functionality of the cardiovascular system.

### 3.2. Basal Atrial Contractility Was Enhanced in Isolated LA After Hypercaloric Diet Exposure

**Figure 2:**
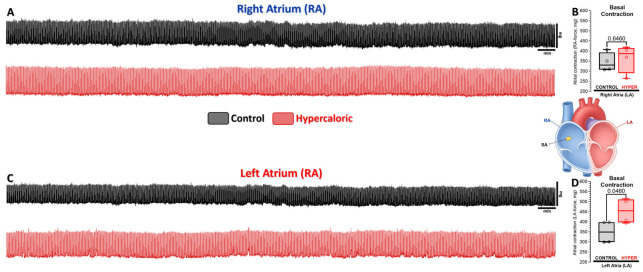
**Basal atrial contractility was enhanced in the left atria** (**LA**) **from rats exposed to 8 weeks of a hypercaloric/hyperlipidic diet.** (**A**) Representative isometric recordings of spontaneous basal contractions from isolated right atria (RA) obtained from 8-week hypercaloric pellet-diet animals and their control (standard diet). (**B**) Quantification of basal RA contractile force. (**C**) Representative traces of electrically stimulated left atrial (LA) contractions from the control (black) and hypercaloric group (red) at week 20. (**D**) Quantification of basal LA contractile force demonstrating increased inotropic activity in the hypercaloric pellet-diet group compared with the control. Schematic illustration depicts isolated RA spontaneous rhythmic activity mediated by sinoatrial node automaticity and electrically stimulated LA preparations. Data are presented as mean ± SD from four and five independent experiments (*n* = 4 and *n* = 5), with data points shown for the control group (black) and hypercaloric group (red) as individual dots in the boxplots. Statistical significance was determined using a two-tailed unpaired Student’s *t*-test, with significance indicated by *p* values. Significance was accepted as indicated by *p* values. RA, right atrium; SA, sinoatrial node; LA, left atrium.

To determine whether a hypercaloric diet alters intrinsic atrial function, basal contractile activity was evaluated in isolated RA and electrically stimulated LA preparations (Figure 2). Isometric recordings demonstrated preserved spontaneous rhythmic contractions in RA preparations from both groups, whereas the LA exhibited greater contractile force under electrical field stimulation conditions (Figure 2A,C). Quantitative analysis revealed that basal contractility of isolated LA was significantly increased (~28%) in hypercaloric animals compared with controls (Figure 2D). These findings indicate that a hypercaloric diet selectively enhances LA inotropic activity, suggesting intrinsic atrial functional remodeling associated with obesity-induced metabolic and cardiovascular dysfunction.

### 3.3. Biphasic Response ATP-Mediated by P1/P2 Purinergic Signaling Was Altered Both in RA and LA

**Figure 3:**
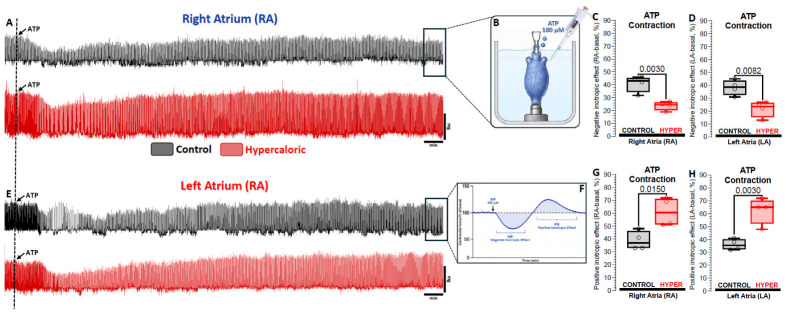
**Hypercaloric/hyperlipidic diet alters ATP-mediated biphasic purinergic responses in isolated right** (**RA**) **and left** (**LA**) **atrial preparations.** (**A**,**E**) Representative isometric recordings showing ATP-induced biphasic purinergic responses in isolated right atria (RA) and left atria (LA) from 8-week hypercaloric pellet-diet animals and their control (standard diet). Extracellular ATP (100 μM) induces an initial negative inotropic effect (NIE), followed by a secondary positive inotropic effect (PIE). (**B**) Schematic illustration of isolated atrial preparation and ATP 100 μM stimulation in an organ bath system. Quantification of ATP-mediated NIE in RA (**C**) and LA (**D**) preparations demonstrating reduced inhibitory purinergic responses in the hypercaloric pellet-diet group. (**F**) Representative schematic tracing illustrating the biphasic ATP-mediated NIE and PIE responses. Quantification of ATP-mediated PIE in RA (**G**) and LA (**H**) preparations demonstrating enhanced excitatory purinergic responses in 8-week hypercaloric pellet-diet animals compared with control (standard diet). Data are presented as mean ± SD from four and five independent experiments (*n* = 4 and *n* = 5), with data points shown for the control group (black) and hypercaloric group (red) as individual dots in the boxplots. Statistical significance was determined using a two-tailed unpaired Student’s *t*-test, with significance indicated by *p* values. Significance was accepted as indicated by *p* values.

ATP-induced inhibitory P1 and excitatory P2 purinergic signaling modulation was evaluated in isolated RA and LA (Figure 3). As shown in the representative recording, ATP (100 μM) induced a characteristic biphasic response in both RA and LA preparations, consisting of an initial NIE, followed by a secondary PIE (Figure 3A,E). The schematic illustrates the biphasic purinergic response mediated by inhibitory P1 signaling and excitatory P2 receptor activation (Figure 3B,F). Quantitative analysis demonstrated that a hypercaloric diet significantly reduced ATP-mediated NIE in both RA and LA preparations (Figure 3C,D). In isolated RA, ATP-induced NIE was markedly decreased (~20%) in hypercaloric animals compared with controls (Figure 3C). A similar reduction (~17%) was observed in ATP-mediated NIE in LA compared with control rats (Figure 3D). By contrast, the secondary positive inotropic response mediated by P2 receptor activation was enhanced in both RA and LA from hypercaloric animals (Figure 3G,H). In RA preparations, a hypercaloric diet increased (~24%) the ATP-induced positive inotropic response compared with controls (Figure 3G). Similarly, isolated LA from hypercaloric rats exhibited enhanced (~29%) PIE following ATP stimulation (Figure 3H). These findings suggest that a hypercaloric diet shifts the purinergic balance toward enhanced excitatory signaling, characterized by reduced inhibitory P1-mediated responses and increased P2-mediated positive inotropic modulation in atrial tissues.

### 3.4. Hypercaloric Diet Promotes Vascular Hyperreactivity and Impairs Purinergic Vasorelaxation

**Figure 4:**
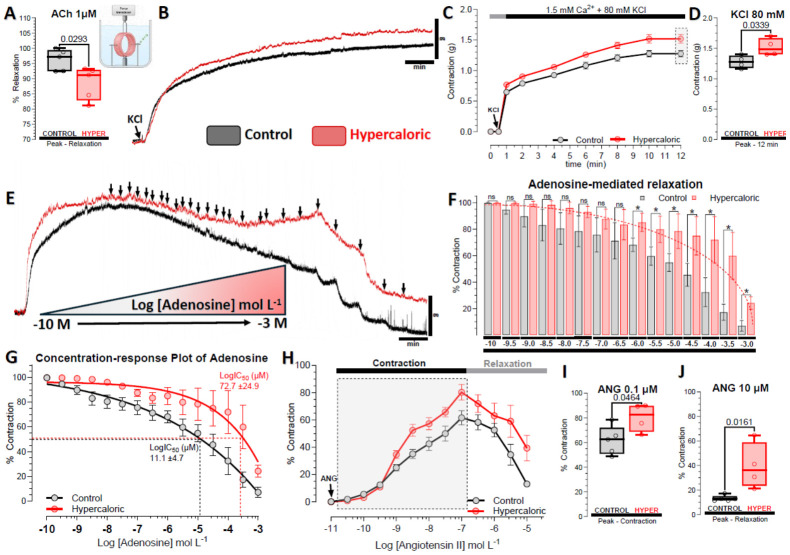
**Hypercaloric/hyperlipidic diet promotes vascular hyperreactivity and impairs adenosine-mediated vasorelaxation in isolated thoracic aortic rings.** (**A**) Acetylcholine-induced (ACh 1 μM) endothelium-dependent relaxation in aorta from 8-week hypercaloric pellet-diet animals and their control (standard diet). Schematic illustration depicts isolated aortic ring preparation mounted in an organ bath system. (**B**) Representative recordings and time-course (**C**) analysis of depolarization-induced vascular contraction stimulated by high-K^+^ solution (KCl, 80 mM). (**D**) Quantification of high-K^+^-mediated peak contraction demonstrating enhanced vascular contractility in 8-week hypercaloric pellet-diet animals compared with controls. (**E**) Representative original traces of cumulative concentration–response plots for adenosine from nano- (0.1 nM, 10^−10^ M) to millimolar (1 mM, 10^−3^ M). Black arrows indicate cumulative adenosine administration, expressed as a percentage reduction relative to phenylephrine-induced contraction. (**F**,**G**) Concentration–response analysis of adenosine-mediated relaxation demonstrating impaired purinergic vasodilatory responses and a rightward shift in hypercaloric animals. (**H**) Concentration–response curve to angiotensin II (10^−11^ to 10^−5^ M) demonstrating enhanced vascular responsiveness in hypercaloric animals. (**I**,**J**) Quantification of ANG II-induced contraction at submicromolar (0.1 μM) and micromolar (10 μM) concentrations. Data are presented as mean ± SD from four and five independent experiments (*n* = 4 and *n* = 5), with data points shown for the control group (black) and the hypercaloric group (red) as individual dots in the boxplots. Statistical significance was determined using a two-tailed unpaired Student’s *t*-test, with significance indicated by *p* values. Significance was accepted as indicated by *p* values. The * symbol denotes *p* < 0.05 (panel (**F**)) for the control group (black) versus the hypercaloric group (red) at 10^−6^ to 10^−3^ M adenosine concentrations; ns, non-significant.

To investigate the vascular effects of a hypercaloric diet, thoracic aortic rings were used to evaluate endothelial function, electromechanical coupling, purinergic vasorelaxation and vasoconstrictor responsiveness (Figure 4). Only aortic rings exhibiting endothelial integrity, defined as ≥80% relaxation to acetylcholine-ACh (1 μM), were included in the analysis. The maximum endothelial-dependent vasorelaxation ACh-elicited was impaired (~93%) in hypercaloric animals compared with the aorta from control rats (Figure 4A). Also, the depolarization-induced vascular contraction elicited by high-K^+^ (KCl, 80 mM) revealed enhanced contractile responses in aortic rings from hypercaloric animals (Figure 4B,C). The hypercaloric diet increased (~28%) KCl-mediated contraction compared with controls (Figure 4D), suggesting enhanced vascular smooth muscle electromechanical coupling and increased contractile responsiveness.

Next, to determine whether a hypercaloric diet induces alterations in aorta P1/P2 purinergic signaling, we evaluated the effect of adenosine in aortic rings. Adenosine is an inhibitory purinergic mediator in vascular tissue [18]; concentration–response curves were constructed to evaluate P1-mediated vascular relaxation. Representative traces demonstrated that cumulative nano-(1 nM) to millimolar (1 mM) concentration–response plots for adenosine progressively reduced vascular contraction in both groups (Figure 4E). However, aortas from hypercaloric diet animals exhibited impaired relaxation responses compared with controls (Figure 4F). The analysis of the IC50 parameter from concentration–response plots confirmed that a hypercaloric diet attenuated adenosine-mediated relaxation (Figure 4G). A rightward shift in the adenosine concentration–response curve was observed, with the LogIC50 increasing from approximately ~11 μM to ~72 μM, suggesting impaired purinergic vasodilatory signaling in the aorta from hypercaloric diet rats. Additionally, the vascular responsiveness to angiotensin II was evaluated as an index of vasoconstrictor sensitivity. Hypercaloric animals demonstrated enhanced contractile responses to angiotensin II compared with controls (Figure 4H). Quantitative analysis revealed significantly increased peak contraction induced by low (0.1 μM), followed by a high (10 μM) angiotensin II concentration in hypercaloric animals (Figure 4I,J). Angiotensin II induced a biphasic concentration–response pattern characterized by increased contraction at intermediate concentrations followed by reduced responses at higher concentrations. Taken together, these results demonstrate that a hypercaloric diet induces vascular hyperreactivity associated with impaired endothelial and purinergic vasorelaxation, enhanced electromechanical coupling, and increased sensitivity to vasoconstrictor stimuli.

## 4. Discussion

The present study demonstrates that 8 weeks of exposure to a hypercaloric–hyperlipidic diet induces a cardiometabolic dysfunction phenotype associated with altered purinergic regulation in both cardiac atria and thoracic aorta (Figure 1, Figure 2, Figure 3 and Figure 4). As expected, and previously reported [37,39,52], a hypercaloric diet promoted obesity-associated metabolic alterations characterized by increased body weight, visceral adiposity, hyperglycemia, insulin resistance, elevated SBP and cardiac hypertrophy. Functionally, these metabolic alterations were associated with increased basal left atrial contractility, reduced ATP-mediated NIEs, enhanced PIEs, impaired adenosine-mediated vascular relaxation, and increased vascular contractile responsiveness. Collectively, these findings support the concept that hypercaloric–hyperlipidic diets induce cardiovascular dysfunction not only through metabolic overload, but also through remodeling of purinergic signaling pathways involved in cardiac and vascular homeostasis. Obesity and metabolic syndrome are strongly associated with increased cardiovascular risk, including hypertension development, endothelial dysfunction, vascular stiffness, cardiac remodeling and CVDs [11,17,32]. Experimental models using hypercaloric or high-fat diets reproduce several features of human cardiometabolic disease, sympathetic overactivation and vascular dysfunction [5,52,53,54]. We observed that a hypercaloric diet increased visceral adiposity and impaired insulin sensitivity, associated with elevated SBP and cardiac hypertrophy, indicating successful establishment of an obesity-associated cardiometabolic phenotype (Figure 1). Similar findings have been reported in animal models exposed to hypercaloric diets, where increased caloric intake and elevated lipid content promote obesity, metabolic dysfunction, and cardiovascular remodeling [55,56]. Human studies also demonstrate that obesity and elevated BMI increase the risk of hypertension, coronary artery disease, atrial dysfunction and cardiovascular mortality [11,12,57].

A hallmark of obesity-associated cardiovascular dysfunction is autonomic imbalance characterized by increased sympathetic nervous system activity [57,58]. Pioneering sympathetic nervous system studies attributed the cardiovascular effects of sympathetic hyperactivity primarily to adrenergic signaling mediated by norepinephrine, α-ARs and β-ARs [57,59]. However, purinergic signaling has emerged as an important mechanism regulating cardiovascular physiology and autonomic neurotransmission [19,21]. ATP is now recognized as a sympathetic cotransmitter released simultaneously with norepinephrine from sympathetic nerve terminals [20,60], where it modulates myocardial function, vascular tone, endothelial signaling and VSMC excitability [18,19]. The function of purinergic receptors is relevant in pathological conditions associated with metabolic stress, inflammation and endothelial dysfunction [61,62,63]. Our results showed an increase in basal LA contractility following hypercaloric diet exposure (Figure 2). Although basal RA spontaneous activity was preserved, LA preparations exhibited significantly enhanced inotropic activity (Figure 2C,D). These findings suggest intrinsic atrial functional remodeling associated with metabolic dysfunction and obesity. This differential response between RA and LA may reflect distinct electrophysiological and functional properties of atrial chambers [64,65]. The RA contains the sinoatrial node and predominantly regulates spontaneous chronotropic activity, whereas the electrically stimulated LA preparation more directly reflects intrinsic myocardial contractile responsiveness independent of pacemaker automaticity [30,66]. Thus, the increased LA contractility observed in hypercaloric animals may indicate early atrial functional remodeling associated with altered excitation-contraction coupling, sympathetic-purinergic imbalance and enhanced intracellular Ca^2+^ handling. Animal models to study experimental obesity and metabolic syndrome models have demonstrated increased atrial oxidative stress, altered β-ARs responsiveness, Ca^2+^ dysregulation and myocardial hypertrophy, which may preferentially affect atrial contractile performance before detectable alterations in spontaneous RA rhythmicity [11,67,68]. In humans, obesity and metabolic dysfunction are also strongly associated with atrial remodeling and increased susceptibility to atrial dysfunction and arrhythmogenesis [67,69].

In the context of atrial dysfunction, ATP induced a characteristic biphasic purinergic response in both RA and LA consisting of an initial inhibitory NIE followed by a secondary excitatory PIE (Figure 3F). Physiologically, ATP is rapidly degraded into adenosine through ectonucleotidases, promoting activation of inhibitory P1 receptors, whereas extracellular ATP directly activates excitatory P2 receptors [23,70,71]. We showed that a hypercaloric diet markedly reduced ATP-mediated NIEs while enhancing ATP-mediated PIEs in both RA and LA preparations (Figure 3C,D,G,H). These findings suggest that a hypercaloric diet shifts the balance between inhibitory adenosinergic-P1 signaling and excitatory ATP-P2 signaling toward an excitatory purinergic phenotype. The physiological balance between inhibitory P1 and excitatory P2 receptor signaling is determined by the coordinated regulation of extracellular nucleotide metabolism and receptor-specific intracellular signaling pathways [20,60]. Following sympathetic nerve stimulation, ATP is coreleased with norepinephrine into the neuroeffector junction, where it can directly activate P2 receptors or undergo rapid sequential hydrolysis by the ectonucleotidases [20,60]. Mechanistically, activation of A1 receptors exerts negative chronotropic and NIEs through Gi-o-coupled pathways, while ATP-mediated activation of P2 receptors causes intracellular Ca^2+^ mobilization and enhanced excitation-contraction coupling [28,66]. Thus, the reduction in NIEs followed by an enhancement of PIEs observed in the atrium from hypercaloric animals, suggests impaired cardioprotective adenosinergic signaling associated with increased excitatory purinergic responsiveness. Similar alterations in purinergic signaling have been described in obesity, DM2 and metabolic syndrome models, where ATP metabolism and purinergic receptor sensitivity were altered [52,67,72]. Hypercaloric and hyperlipidic diets, obesity and hyperglycemia are associated with chronic inflammation and increased extracellular ATP release from endothelial cells and sympathetic terminals [1,15,73,74]. Elevated extracellular ATP may sustain activation of P2 receptors and contribute to cardiac dysfunction, inflammation, oxidative stress, and abnormal Ca^2+^ signaling [28,73,75]. By contrast, adenosine-mediated P1 signaling is considered cardioprotective due to its anti-inflammatory, anti-adrenergic and anti-arrhythmogenic properties [63,76]. The reduction in ATP-mediated NIE may reflect impaired adenosinergic signaling during hypercaloric diet-induced metabolic stress.

At the vascular level, to determine the vascular functional effects of a hypercaloric diet, we evaluated endothelial function, electromechanical coupling, purinergic vasorelaxation and vasoconstrictor responsiveness. Hypercaloric diet promoted marked vascular hyperreactivity characterized by impaired acetylcholine-mediated endothelial relaxation, increased KCl-induced contraction, attenuated adenosine-mediated vasorelaxation, and enhanced angiotensin II responsiveness (Figure 4). Endothelial dysfunction is considered an early hallmark of obesity-associated cardiovascular disease and is closely associated with impaired NO bioavailability, oxidative stress, inflammation, and VSMC dysfunction [2,77,78]. We observed an impaired acetylcholine-ACh-mediated relaxation that suggests reduced endothelial NO signaling in hypercaloric animals, consistent with previous reports, both in mice and humans, demonstrating endothelial dysfunction in obesity and metabolic syndrome [79,80,81]. The enhanced contraction induced by high-K+ further suggests increased VSMCs electromechanical coupling and enhanced Ca^2+^-dependent contractility. Also, high extracellular K^+^ depolarizes the plasma membrane and activates voltage-dependent L-type Ca^2+^ channels (Cav1.2), promoting intracellular Ca^2+^ influx and vascular contraction [79,80]. Thus, the increased high-K^+^ responsiveness observed in hypercaloric animals may indicate altered VSMC excitability and Ca^2+^ handling associated with obesity-induced vascular remodeling. Similar alterations in contractility have been reported in high-fat diet and diabetic models, where VSMCs exhibit increased contractile sensitivity and impaired Ca^2+^ homeostasis.

Next, our study revealed an impaired adenosine-mediated vasorelaxation observed in hypercaloric animals (Figure 4F,G). Adenosine is an important inhibitory purinergic mediator that induces vascular relaxation mainly through activation of A2A-B receptors and reduction in intracellular Ca^2+^ signaling in VSMCs. The rightward shift observed in adenosine concentration–response curves indicates reduced purinergic vasodilatory sensitivity in hypercaloric animals (Figure 4F,G). Similar findings have been reported in diabetic and obese rodent experimental models, where impaired adenosinergic signaling contributes to endothelial dysfunction, reduced vascular compliance and impaired tissue perfusion [82,83,84]. Human studies also indicate that purinergic vasodilatory pathways are altered in DM2 and obesity, contributing to vascular dysfunction and increased cardiovascular risk [15,85,86]. Eight weeks of a hypercaloric diet also increased vascular responsiveness to angiotensin II, reinforcing the concept of vascular hyperreactivity during obesity-associated metabolic dysfunction (Figure 4H–J). Increased angiotensin II responsiveness has been consistently described in obesity and hypertension models and may contribute to elevated peripheral resistance and increased SBP [87,88,89]. Interestingly, angiotensin II produced a biphasic concentration–response profile characterized by increased contraction at intermediate (submicromolar) concentrations followed by partial reduction at higher concentrations (micromolar) [90,91,92,93]. Thus, the attenuation of adenosinergic vasodilatory signaling may reduce compensatory anti-contractile mechanisms, favoring enhanced angiotensin II-mediated vascular reactivity during hypercaloric diet-induced metabolic dysfunction. This imbalance between inhibitory purinergic relaxation and vasoconstrictor responsiveness may contribute to endothelial dysfunction, vascular stiffness, and elevated SBP observed in obesity-associated CVDs.

Collectively, the present findings support the concept that hypercaloric–hyperlipidic diets promote cardiovascular dysfunction through integrated metabolic, autonomic, and purinergic mechanisms. The shift from inhibitory cardioprotective adenosinergic-P1 signaling toward enhanced excitatory ATP-P2 signaling may contribute to atrial dysfunction, vascular hyperreactivity, endothelial impairment, and increased CVD risk associated with obesity and metabolic disorders. Although the present study demonstrates significant alterations in atrial and vascular purinergic responses following hypercaloric diet exposure, some limitations should be acknowledged. The proposed mechanisms involving altered P1 and P2 receptor signaling are based on functional pharmacological responses and were not directly confirmed by molecular analyses. The expression of purinergic receptor subtypes, ectonucleotidases (CD39/CD73), intracellular Ca^2+^-handling proteins, inflammatory mediators, oxidative stress markers, and downstream signaling pathways was not evaluated. Another limitation of the present study is that only male rats were evaluated. Sex hormones influence cardiovascular physiology, metabolic regulation and purinergic signaling, and the present findings cannot be directly extrapolated to females [94]. Future studies should determine whether a hypercaloric diet induces similar alterations in atrial and vascular purinergic signaling in female animals and evaluate the contribution of sex hormones to obesity-associated cardiovascular remodeling. Therefore, the molecular mechanisms discussed should be interpreted as biologically plausible hypotheses supported by previous experimental studies rather than direct conclusions from the present work. Together, these findings reinforce purinergic signaling as a potential therapeutic target in obesity-associated cardiovascular dysfunction and hypertension. Future investigations combining molecular, biochemical, electrophysiological, and pharmacological approaches will be important to determine how a hypercaloric diet remodels purinergic signaling and intracellular Ca^2+^ homeostasis in the heart and vasculature.

## 5. Conclusions

Chronic hypercaloric–hyperlipidic diet induced obesity-associated cardiometabolic dysfunction characterized by increased visceral adiposity, hyperglycemia, insulin resistance, elevated SBP, and cardiac hypertrophy. These metabolic alterations were accompanied by significant functional remodeling of cardiovascular purinergic signaling, including reduced ATP-mediated inhibitory responses and enhanced excitatory responses in atrial tissue, as well as impaired adenosine-mediated vasorelaxation and increased vascular contractility. Together, these findings indicate that a hypercaloric diet disrupts the physiological balance between cardioprotective adenosinergic-P1 and excitatory ATP-P2 signaling, contributing to atrial dysfunction, vascular hyperreactivity and obesity-associated cardiovascular remodeling. This study provides novel functional evidence supporting purinergic signaling as an important mechanism linking metabolic dysfunction to cardiovascular impairment and highlights this pathway as a promising therapeutic target for obesity-associated cardiovascular diseases.

## Data Availability

The data that support the findings of this study are available from the corresponding author, Guilherme H. Souza Bomfim upon reasonable request.

## Abbreviations

The following abbreviations are used in this manuscript:

ACh	Acetylcholine
α-ARs	Alpha Adrenergic Receptors
ANG II	Angiotensin II
ATP	Adenosine Triphosphate
BMI	Body Mass Index
β-ARs	Beta Adrenergic Receptors
Ca^2+^	Calcium
Ca_v_1.2	Voltage-dependent L-type Ca^2+^ Channels
CVDs	Cardiovascular Diseases
DAG	Diacylglycerol
DM2	Diabetes Mellitus Type 2
IP3	Inositol Triphosphate
ITT	Intravenous Insulin Tolerance Test
KHN	Krebs–Henseleit Nutrient
KITT	Plasma Glucose Disappearance Rate
LA	Left Atria
NIE	Negative Inotropic Effect
NO	Nitric Oxide
Phe	Phenylephrine (1 μM)
PIE	Positive Inotropic Effect
PLC	Phospholipase C
RA	Right Atria
SBP	Systolic Blood Pressure
SNS	Sympathetic Nervous System
VSMCs	Vascular Smooth Muscle Cells
